# Thinking Through Historical Analogies: A Longitudinal Analysis of Sense-Making During the Pandemic

**DOI:** 10.1007/s12124-025-09942-3

**Published:** 2025-10-21

**Authors:** Brady Wagoner, Lisa Herbig

**Affiliations:** 1 Department of Psychology, University of Copenhagen, Copenhagen, Denmark; 2 Department of European Studies, University of Amsterdam, Amsterdam, Netherlands; 3 Communication and Psychology, Aalborg University, Aalborg, Denmark; 4 Department of Psychology, Oslo New University College, Oslo, Norway

**Keywords:** Historical analogies, Covid-19 pandemic, Conspiracy theories, Longitudinal data, Dialogical analysis

## Abstract

Historical analogies (HAs) are cultural tools for making sense of a current situation by drawing comparisons to a past event. Their use in communication and understanding can be observed since ancient times and in societies around the world, especially in times of crisis. The present paper explores the role they played in people’s everyday thinking during the COVID-19 pandemic, including what HAs were used, how they changed over time, who used them, and to what effect. To do this it draws on a longitudinal project in Germany that included a national representative survey (*N* = 1080) and follow-up interviews (*N* = 38) done at three data collection points. The interviews were coded for HAs, the results of which are presented in three steps: first, an overview of the HAs used and their change over time is given; second, characteristics of the most frequent users are outlined; and third, a case study of the most frequent user is presented. The article highlights the everyday use of HAs by people through time, their social distribution between majorities and active minorities, their link to conspiracy mentality and their personal psychological functions.

## Introduction

During an interview in September 2020, a middle-aged German woman expressed her concerns about the COVID-19 pandemic in these words:

The fact that people will no longer have work (…) that’s what I’m most afraid of, because (…) if they really have no more work, they‘ll vote radically (…) as in 18/19, after the First World War. The fact that people then had to continue to make large reparation payments to France (…). Only through that could a party like the NSDAP become big. And I’m actually a bit afraid of that, because that’s the breeding ground for extremism, which is what’s being done here now.

The quotation makes a strong *historical analogy* (HA) between the pandemic situation and the situation in German after WWI, which led to the rise of the Nazis. Not only does the HA help her to *make sense* of the disorienting situation she is now living through, but it also *predicts* for her what will come in the future on the basis of what has happened in the past. Furthermore, it is being used as a *rhetorical device* to subtly make the argument that implementing strong government measures to contain the virus are misguided and will have dire political consequences. We will discuss below how it also serves to define the roles of different actors and fulfils a personal psychological function. While this is a striking example, the use of HAs and these functions it serves are familiar to us as part of everyday communication. Yet little has been written to explore how they operate through time in people’s everyday lives, particularly during times of crisis and change. Instead, most research tends to study HAs as the appear in public discourse or as part of a cognitive function in a controlled experimental setting, and thus misses people’s sense-making as it is situated their communication and action with others.

This article asks the general question “What role did HAs play in the way that people made sense of the COVID-19 pandemic in Germany?” More specifically, it explores: what HAs were used, how did they change over time, who used them and to what effect. To answer these questions, an open-access longitudinal dataset about the German public’s response to the COVID-19 pandemic was analyzed (Herbig et al., 2022; Jensen et al., 2021). Germany is a particularly suitable context for this research, given that its recent history of two dictatorships continues to loom large in its collective memory. The dataset was not collected with HAs in mind, but they frequently appeared in answers to questions about a variety of different topics. After reviewing the literature on HAs (esp., during COVID-19) and the present dataset, the analysis of HAs proceeds in three steps: *First*, an overview of what HAs were used and how they changed over time is presented for the entire sample; *second*, the characteristics of the most frequent users of HAs in the sample are outlined; and *third*, a case study of the most frequent user is given to illustrate how HAs operate in a person’s psychological system, becoming deeply internalized frames for making sense of the world. The paper ends with a reflection on the social distribution of HAs (between majorities and active minorities), their link to conspiracy mentality and their personal psychological functions.

## Functions and Effects of Historical Analogies

Sudden crises are followed by attempts to make sense of the ambiguous and uncertain situation. One main way in which this is done is by creating analogies, which help to make the unfamiliar familiar within existing frames of reference (Moscovici, 2000). HAs are a particular species of this process, in which explicit parallels are drawn between a present event and a past event that is typically already potent within a group’s collective memory (Halbwachs, 1980). These tend to be *within-domain* (literal) comparisons rather than *between-domains* (metaphorical) comparisons, though both play a role. For example, comparing the COVID-19 pandemic to the 1918 pandemic is *within domain*, whereas NY governor Andrew Cuomo’s statement that “Ventilators are to this war what bombs were to World War Two” is *between-domains.* The latter example builds on the widely used but problematic metaphor of being at war with a disease (Sontag, 1991; Wagoner & Herbig, 2023), while the former does not presuppose a metaphorical linkage. Building mainly on within domain HAs, Ghilani et al. (2017) provide a helpful review of what is known about them in terms of four interrelated effects: (1) representing a current situation, (2) defining roles of current actors, (3) making decisions, and (4) persuading others.

### Representing a Current Situation

HAs shape how a current situation is seen and felt by representing it through a certain frame. When a current situation is complex, ambiguous and uncertain, HAs work to simplify and organize it into a coherent narrative. When news of the COVID-19 was first reported in January 2020, HAs to the 2002–2003 SARS outbreak dominated the Flemish media, striking a reassuring tone with the public (De Ridder, 2020). However, as the COVID-19 death toll surpassed SARS in February and March a sentiment of alarm began to creep in and a wider range of HAs were used, such as ‘the Plague,’ ‘the Spanish Flu’ and ‘AIDS’. The 1918 pandemic was itself only dimly held in collective memory before COVID-19 (transmitted mainly by historians and epidemiologists), despite its killing 50–100 million people and global impact more generally (Spinney, 2018). In 2020, however, it quickly returned with vengeance, becoming a powerful HA to understand COVID-19 (Erll, 2020). Looking at the broader literature, WWII has been a particular persistent HA in American and European political discussions and is probably the most researched HA. In the US, new military interventions are often justified by making explicit links to it, but it was also used to justify strict measures during COVID-19. In contrast, the Vietnam War—a war that was originally itself justified through a WWII HA—is a potent event in Americans’ collective memory that represents the (moral) hazards of war. In an experimental study, Gilovich (1981) showed that Americans will assess a hypothetical military intervention more positively when they are merely incidentally reminded of WWII versus being reminded of the Vietnam War (see also third effect below). Like the changing HAs to COVID-19, so too do HAs for wars shift with events. Both the Gulf War and Iraq War were initially justified by WWII HAs, but as the Gulf War progressed Vietnam became the dominant HA as national sentiments shifted (Shuman and Rieger, 1992), and for the Iraq War (2003–2011) democratic Senator Kennedy in 2004 was already commenting, “Iraq is George Bush’s Vietnam.” More recently, analogies were widely made between the US’s blotched withdrawal from Afghanistan in 2021 and its withdrawal from Vietnam.

### Defining Roles

In narrativizing events roles need to be assigned to actors. HAs are here a powerful tool in that they provide clear sets of the heroes, victims, and villains. If the Iraq War is like WWII, then Saddam Hussein is like Hitler, and by implication the US is the hero, saving the day once again. By contrast, the Vietnam War analogy casts the US in the role of villain. Furthermore, ascribing roles works to define group boundaries and shape social identities. This ‘we’ can then be the locus of collective emotions, such as pride for WWII and shame for the Vietnam War. During the COVID-19 lockdowns, HAs were made to WWII with the goal of generating group solidarity and joint efforts in the face of societal challenges. One US newspaper article was published with the title “Coronavirus comparisons to World War II are rampant. Are we ready to be greatest generation?” (Hinds, 2020). At the same time, protestors against measures to contain the virus used HAs from WWII to cast the government into a tyrannical role (like Hitler and National Socialism) and themselves as fighters for liberty and democracy, like the non-violent anti-Nazi activist Sophie Scholl who was executed by the Nazis in 1943. Similarly, protestors saw the vaccination pass requirement as a device to segregate them as a group, making an audacious analogy to the “yellow star” worn by Jews under the Nazis.

### Making Decisions

HAs can contribute to future oriented reasoning, to understand what is to come and what to do about it now. While current situations are open to action, past situations are closed (only their interpretation is open). The narrative structure provided by the past event presents a limited range of possible future scenarios and the appropriate actions to reach the most desirable outcome. The framing of potential military conflicts in terms of WWII and Vietnam make the decision to enter them or not a straightforward choice. This will often also include counter-factual reasoning: ‘What would the world be like now if the US had not intervened to stop Hitler?!’ In relation to COVID-19, the women in the quotation at the start reasons that implementing restrictions will create economic instability and lead to political radicalization, just as it did in post WWI Germany, leading to Hitler. With the use of this HA, it is clear the restrictions need to be lifted to avoid this potential future catastrophe (see also fourth effect). This HA was widespread among groups protesting government restrictions, such as the *Querdenker* (lateral thinkers) in Germany. Their identity as fighters against oppression (see second effect), comes from the idea that there will be tyranny in the future if citizens do not actively resist the government’s overstep of its powers which threatens individual freedoms. This kind of resistance HA was much more prominent among people in the East (former GDR) than in West Germany, highlighting their differing historical experiences (Coenen et al., 2024).

### Persuading Others

HAs are powerful rhetorical devices for *convincing others* through an appeal to “historical truth”. They give “authority, legitimacy and feasibility” (Ghilani et al., 2017, p. 281) to one’s arguments by subtly eliciting powerful images and affective reactions. It is thus not surprising that German groups protesting COVID-19 restrictions heavily used familiar Nazi HAs. In addition to the examples already given above, a Munich man posted an image of Auschwitz’s gate with ‘Impfung macht frei’ (vaccination makes free) in the place of ‘Arbeit macht frei’ (work makes free). On the other side, wartime analogies were convincing tactics in fostering unity and compliance with pandemic restrictions. However, in appealing to others there is always a risk of misjudgement that can lead to backlash if HAs are perceived as inappropriate or manipulative by an audience. Comparisons between the COVID-19 pandemic and the HIV/AIDS pandemic were strongly resisted by gay advocacy groups because AIDS, unlike COVID-19, was met with a slow government response due to it disproportionately affecting stigmatized groups (viz., gay and African American groups) (Catlin, 2021).

The above review is based mainly on an analysis of public discourse and a few psychology experiments. It should also be noted that the focus is mainly on political HAs, such as wars and protests. From this starting place, there is little attention to how they operate as part of people’s concrete meaning making in daily interaction with others and for making decisions that matter for their lives. Thus, most research misses the reflective and critical use of HAs, how HAs are also rejected and circumscribed, what personal functions HAs serve and how they change. In short, there is a gap in understanding how HAs function within people’s everyday thinking through time. This paper aims to fill this gap through a detailed, longitudinal and triangulated analysis of people’s everyday use of HAs to make sense of the COVID-19 pandemic.

## Methods

### Sample and Recruitment

This article draws on data from the *Viral Communication* project, which applies a mixed-methods research approach to investigate public attitudes, beliefs, and behaviors during the COVID-19 pandemic in Germany (see Jensen et al., 2021; Herbig et al., 2022). The project began with a nationally representative online survey conducted in November 2020, which captured a broad spectrum of opinions on pandemic-related topics, such as trust, compliance, information seeking, and vaccination. Of the more than 1,000 respondents, 278 expressed willingness to participate in follow-up interviews. From this pool, 40 participants were purposively sampled to ensure diversity in age, gender, and socioeconomic status (SES), as well as variation in trust levels, migration background, vaccination attitudes, and compliance behaviors.

These participants took part in a longitudinal qualitative interview study comprising three rounds of semi-structured interviews conducted between December 2020 and September 2021. Two participants dropped out after the first round, resulting in a final longitudinal dataset of 38 individuals. Each participant was interviewed at intervals of approximately every 3–4 months via telephone or Zoom, with interviews averaging 40–45 min in length. The interview protocols were designed to elaborate on key survey findings from the survey and probe emerging issues such as vaccine hesitancy, conspiracy beliefs, and trust in scientific and political authorities. Interview questions were updated at each round to reflect the evolving pandemic context but the interviews were kept to around the same length of time.

The study followed a semi-structured format with fixed thematic blocks, allowing for consistent tracking of key topics (e.g., information sources, protective behaviors, trust in institutions, and outlooks on the future), while remaining open to participants’ unique narratives and experiences. The interviews began with a broad reflection on recent developments, followed by targeted questions linked to prior survey responses. All interviews were conducted in German, audio-recorded with participants’ consent, and transcribed using an intelligent verbatim approach. Transcripts were anonymized and lightly edited for clarity, with indicators of tone (e.g., laughter, irony) preserved.

Ethical approval was granted by the Ethics Committee of Sigmund Freud University, and participants provided informed consent for full anonymized transcripts to be made publicly available. The complete dataset, including interview transcripts (in German), SPSS demographic files, and the full interview guides in German and English, is openly accessible via Zenodo: 10.5281/zenodo.5556052. Further details on sampling criteria and procedures are also available in Herbig et al. (2022).

### Coding Historical Analogies

This study examines how individuals used historical analogies (HAs) to make sense of the COVID-19 pandemic in Germany. We define HAs as explicit comparisons between present events and past events. Operationally, we coded as HAs only those utterances that both referenced a specific past event or circumscribed period of time, and drew an explicit comparison to some aspect of the COVID-19 pandemic, including its causes, effects, actors, policies, or anticipated future outcomes.

This strict interpretation of HAs excluded other kinds of references to the past, such as reference to a generalized “past” rather than concrete historical moment. For example, one participant mentioned a saying they heard on social media: “In earlier times, during serious crises, the leaders - the heads of state - were sacrificed to the gods” which was followed by “one should also leave no stone unturned in today’s crises.” (02_2), implying today’s leaders should be likewise sacrificed. There is an allusion to a mythicized “earlier time” here but not a specific historical event or period.

We also excluded statements arguing that a present situation is not like the past. While such utterances contain a comparison, their function is to distance the current crisis from a historical precedent. Several participants, for example, said that people were acting as if it was “the plague” when in fact it is, as one participant put it, “a disease where mainly people over 80 die, and where most people don’t even notice when they’re infected.” (38_1). Rather than framing the present through the lens of the past, this historical reference serves to critique perceived overreactions to policies and people. In some cases, this could be understood as participants implicitly voicing other people’s HAs in order to ridicule and undermine them.

The dataset was inductively coded by two native German speakers in three phases. First, a comprehensive list of keywords (see Appendix Table A) related to major historical events was used to flag relevant passages in the interview transcripts for consideration. Second, a random sample amounting to 10% of the full corpus was manually reviewed line by line to ensure that analogies expressed in less explicit language were not missed. Third, we employed a large language model (ChatGPT-4o) to check whether any historical analogies had been overlooked. This step yielded just one additional analogy, suggesting that our hybrid approach was highly effective in ensuring thorough and reliable coding.

Our coding of different HAs resulted in two superordinate categories: Medical and political. Medical included mainly references to ‘previous pandemics’ such as the Spanish Flu, Swine Flu, the Plague, Ebola and Bird Flu (listed from most to least frequent), and ‘medical scandals’ such as Contergan (medication linked to birth defects) and the Swine Flu vaccine (which caused 25 deaths and was found to be linked to Guillain-Barre Syndrome). Although all these medical scandals happened decades ago and led to further regulatory oversight, they continue to be present in collective memory and cause distrust. Political HAs were dominated by references to the Second World War (including National Socialism) and the German Democratic Republic (GDR). In addition to these main codes, there were also less frequently mentioned HAs (i.e., appearing 1–2 times in the dataset). These were coded as ‘other’ within either the political or medical domain. Some examples of these ‘other’ HAs in politics are the 9/11 attacks, the First World War, and the so-called “refugee crisis” of 2015. Examples of the different codes can be found in Table 1 below.

**Table 1:** Overview of the applied codes

Category	Code	Example
Medical	Previous Pandemics	*“Someone who is completely against (vaccination told me) “You’re not allowed to vaccinate in pandemics“ (because it promotes mutations). But (…) what else should we do? Do we want to wait until millions of people die like in the Spanish flu*,* I think there were 50 million dead. Do we really want to take that chance?*” (27_R2)
	Medical Scandals	*“You saw it a bit back then with the swine flu. The government bought a vaccine*,* spent a lot of money*,* and in the end*,* they weren’t properly tested and had to be destroyed. And I think the same thing could happen again now.”* (16_R3)
	Other Medical	*“It’s obviously not officially desired – by the RKI or similar institutions – to gain any insight by conducting more autopsies. And to me*,* that reminds me of the Middle Ages*,* when it was frowned upon in Germany*,* for religious reasons*,* to dissect bodies. That was something they did in the Arab world*,* which is why medicine there was so advanced at the time*,* right? All the well-known doctors came from the Arab cultural sphere. Because they did that there*,* and here*,* people who wanted to do such things were burned as witches.”* (39_R3)
Political	World War II/National Socialism	*“But I find something like that extremely undemocratic. And I also suspect that secret agreements are being made*,* so to speak. Because what other effect could it have? It’s a kind of Hitler-Stalin pact*,* what they’re doing.”* (39_R2)
	German Democratic Republic	*“And for me*,* that’s already a kind of surveillance*,* a way to monitor citizens. It’s almost like back then in the GDR*,* where the ‘block warden’*,* so to speak*,* would check whether I was going to work or not.”* (25_R1)
	Other Political	*“In recent years*,* it’s always been the case that whenever something happened*,* higher standards were introduced for such concepts and events. I’m thinking of the Love Parade disaster in 2009/10*,* after which safety regulations for large events were significantly tightened. (…) I can imagine that many organizers no longer want to take on that kind of risk and responsibility.”*

## Results and Analysis

### Overview and Changes of HAs

The frequencies of the different codes across the three time points are presented in Table 2. Several trends stand out as noteworthy: First, the most frequent HAs (43% of the total) refer to previous pandemics, which is somewhat unsurprising given the straightforward and direct way these can be compared to the COVID-19 pandemic. However, the second most frequent HA (27% of the total) was ‘WWII/national socialism’ which at first sight has very little to do with the pandemic. As mentioned above, this was a common HA used by those protesting the government’s COVID-19 measures and in our sample was used by those that were sympathetic with this movement. Second, the overall frequency of HAs decreases across the three time points: 42 instances in round 1, 28 in round 2, and 12 in round 3. This can be explained by the pandemic situation being still rather disorienting in the first round, such that participants were reaching for whatever sense making devices they could get a hold of. By the third round, the pandemic situation was beginning to be normalized, even if there were new developments. Third, the relative importance of medical and political HAs reversed over the three time points. In the first round, medical HAs make up 61.9% of the total compared with 38% for political HAs, in round 2 the percentages are roughly equal, and in the third round 66% are political HAs compared to only 33% being medical (although there is a much smaller number of HAs used in the third round). This trend can be understood in that political HAs tend to be employed by those who resisted the pandemic restrictions, which remained in place beyond our study. This brings us to the question of the distribution of HA use between participants in our sample.

**Table 2:** Overview of coded historical analogies over time

Category	Code	Round 1 (%)	Round 2 (%)	Round 3 (%)	Total (%)
Medical (SUM)		26 (61.9)	14 (48.3)	4 (33)	44 (53)
	Previous Pandemics	19 (45.2)	14 (48.3)	3 (25.0)	36 (43.4)
	Medical Scandals	6 (14.3)	0 (0)	0 (0)	6 (7.2)
	Other Medical	1 (2.4)	0 (0)	1 (8.3)	2 (2.4)
Political (SUM)		**16 (38)**	**15 (51.7)**	**8 (66)**	**39 (47)**
	WWII/NS	10 (23.8)	8 (27.6)	4 (33.3)	22 (26.5)
	GDR	1 (2.4)	2 (6.9)	2 (16.7)	5 (6)
	Other Political	5 (11.9)	5 (17.2)	2 (16.7)	12 (14.5)
Total		**42 (51.2)**	**28 (34.1)**	**12 (14.6)**	83 **(100)**

### HA User Characteristics and Frequences

The next step in the analysis was to look at who was using HAs. Table 3 shows that the majority of participants either do not use HAs at all (10 participants or 26.32%) or do so infrequently (e.g., 11 participants or 28.95% only use them a single time). On the other extreme, one participant used them 19 times across the three interviews, which is 22.9% of the HAs within the entire sample. The two extreme users were both middle-aged with some higher education and were highly critical of the pandemic restrictions. What really sets them apart from the others was their degree of conspiracy mindedness. As part of our questionnaire, we included a conspiracy mindedness scale (Bruder et al., 2013) that was adapted to describe the pandemic situation. There were four items that asked for participants’ level of agreement with statements like “Politicians have been hiding their true motives for how they have handled the Coronavirus (COVID-19) situation” and “The Coronavirus (COVID-19) situation has provided an excuse for government agencies to closely monitor all citizens.” The top HA users had an average aggregated score of 4.38 (S.D.=0.18) on this 7-point scale, compared at an average of 2.77 (S.D.=1.65) for the other interviewees. In the next section we will look in-depth at the most frequent HA user to illustrate how HAs operate within a complex single case.

**Table 3:** Overview of the use of historical analogies by participants

# Historical Analogies		# of Participants		% of Participants
0	10		26.32	
1	11		28.95	
2	6		15.79	
3	6		15.79	
4	2		5.27	
5	1		2.63	
10	1		2.63	
19	1		2.63	

### Case Study Analysis

For this case study, we conducted a dialogical analysis (Aveling et al., 2014; Gillespie & Cornish, 2010; Wagoner et al., 2011), which involves identifying the internal and external positions or voices a person speaks from and then analysing their relationship to one another. The analysis includes positioning on macro (societal), meso (family) and micro (individual) levels for a nuanced look at collective memory (Cordonnier et al., 2022). Given the longitudinal nature of the data, we can also explore how this matrix of positions changes through time. The woman we will analyse is middle age, of lower socio-economic status, and living in southern Germany.

In terms of HAs, she persistently frames the pandemic in terms of National Socialism in all three interviews. This HA is rhetorically used to criticize the Covid-19 measures, but it is also an apt expression of her worldview, characterised by extreme distrust in authorities which only increases across the three time points. In an explicit HA between current politicians and those under National Socialism she says, “these little men, like back then, they get power now”. At several points in the interviews, this extreme distrust manifests itself in in terms of conspiracy beliefs. Her representation of the structure of society and her own position as an outgroup is also found in research with different conspiracy theorists, although it should be noted that the ‘evil elites’ are not fully hidden for her (Franks et al., 2017; Pahuus et al., 2024). She describes how politicians, media and scientific institutions pursue their own malevolent interests. This involves instilling fear, ignorance and intolerance in ‘the people,’ who she sees as otherwise acting reasonably. In contrast to the majority of society, she positions herself as part of an outgroup of ‘critical thinkers’ who seek out alternative sources of information (esp., in social media links from ‘other critical thinkers’). She is also positioned as an outsider by other people, who she feels have hostilely “observed and antagonized” her for her noncompliance.

Beyond these societal positions, her use of HAs is tightly intertwined with her interpersonal relationships. Of particular importance is her family, who she positions among the critical thinkers, saying “we question everything in our family”. As observed by Halbwachs (1992), statements like this function to create an affective identity within a family through which it transmits its memories through time. She says her parents taught her “not (to be) obedient to the authorities”. Most revealing of all is her grandfather, whose voice consistently warns her of the dangers of National Socialism: “My grandfather always says, ‘it starts like 33’”. We have no way of checking whether he was in fact part of the resistant against the Nazis, but previous research has found that in the third generation after the war, Germans tend to reconstruct the story of their grandfather from the position of Nazi into someone fighting against the Nazis (Welzer, 2005). This rewriting of earlier generations memory is a kind of compromise formation between the horrors of Nazism that they have diligently learned in their formal history education and the personal relationship they have to a beloved family member.

Other family members like her brother, uncle and son also fit the label of ‘critical thinkers,’ but her daughter and son-in-law do not because she says, they “have been hit by all the media propaganda” (like ‘the people’ in the above general representation of society). They likewise position her as being ignorant and right-wing in her beliefs: She says her son-in-law “dismisses everything that is not corona compliant as radical right-wing” and “insulted my intelligence… (saying) I always believe what’s on the internet”. Her daughter is characterised as a “total vaccination advocate” who asks her, “Mum, do you really want to radicalise yourself?” By the third interview she has become more estranged from them, only mentioning her daughter once in response to a question about the “biggest challenges” of the pandemic.

Her distrust in government and proud family tradition of standing up to authority create an appeal to various groups protesting the COVID-19 restrictions. Already in the first interview she says: “I’ll probably have to join (the *Querdenker*), especially now that they’re being watched by the Office for the Protection of the Constitution (*Verfassungsschutz*), because basically it’s a peaceful movement.” By the third interview, her tone has become more decisive about joining the protests. Talking about a woman who gave a speech against vaccination at a protest she says:

And this woman is also networked, and wants to start in the area here and I’m going to (…) see that I get her email and then I’m going to network with her. Something has to happen. My grandfather was already in the underground against the Nazis, so I think it’s up to us in the family not to let others dictate to us what to do and instead to think for ourselves. I’ll network and see what comes of it, right?

**Fig. 1:**
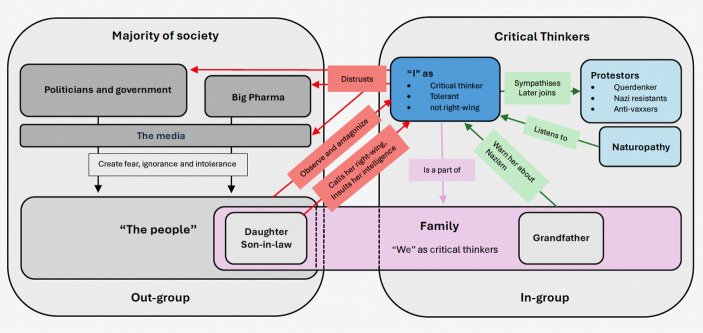
Overview of positions and their relationships

We know from a fourth interview with her (only conducted with the six anti-vaxxers in our sample) that she in fact does join. Much of the tension with her daughter and son-in-law came from their associating her with the “radical right-wing” which she vehemently denies in the first interview: “I’m guaranteed to be anything but right-wing!” In the third interview, she is aware that these protesters tend to be right-wing but justifies the association: “Even if a right-winger runs along against masks, against state arbitrariness, against quarantine, against compulsory vaccination, then I personally do not care, because it is about the cause.” After attending the protest in the fourth interview, she is asked about neo-Nazi elements in the movement for which she responses “If a Nazi says ‘2 + 2 equals 4,’ it is not wrong” (Fig. 1).

Having mapped out the different positions from which she speaks, their relationships and change over time, we are now able to better understand her use of HAs (esp. regarding National Socialism) to frame various topics concerning the pandemic. She was the one who expressed fears of rising extremism in the quotation at the beginning of the article, where she makes the HA to the situation “in 18/19, after the First World War” which led to the Nazis. On the topic of facemasks, she makes a HA to Germany’s earlier intolerance and persecution of minorities:

“Well, what I feel: hysteria, fear, denunciation, panic. Well, not myself, but I experience it in my fellow human beings. Very, very bad. (…) Customers (not wearing a face mask) are insulted in the shop (…). So, it sounds a bit like my grandfather told me during the persecution of the Jews: (People said) ‘Don’t buy from the Jews, don’t buy this and that’, you know? Well, it scares me. Actually, what scares me is how people treat each other, the lack of tolerance.” (R1).

Again, we see the majority structure of society discussed above and her own position as standing outside it. Similarly on the topic of quarantine, she describes how neighbours now spy on and report each other as they did before. As with the previous quote, the voice of her grandfather is explicitly present warning her of the potential dangers. In the quote, she tells the story of a locally famous soccer player that was supposed to quarantine:

He tested positive (…) he went out for air at ten o’clock at night. He was reported by some neighbour, although he didn’t endanger anyone, did he? And then he goes out and is reported by someone who sits at the window with binoculars behind him and looks with them and makes sure that no one else is doing anything, right? (…) It reminds me, my grandfather always says, ‘It starts like 33’.

Her use of the HA here is interesting in comparison to another participant who lived in the former GDR. Both express the same high suspicion of surveillance by government and fellow citizens but express it through different HAs. For one the suspicion is transmitted through family memory of the Nazis era, while for the other it is a lived experience of being monitored by the “block warden” who he says, “would check whether I was going to work”. The latter is a generic memory of the GDR which sums up different experiences into a composite image (Halbwachs, 1992). This participant remained unvaccinated to the end of our study, with serious consequences for his work and family.

With regards to vaccination, she is in favour of alternative medicine and mentions her naturopath as well as other alternative doctors. She places them in the camp of critical thinkers as opposed to medical institutions and experts who tend to follow the government and big Pharma. For our purposes, the most important HA for this topic is the one she uses to cast the unvaccinated as persecuted victims:

“I don’t like it at all when they say (there will be) an indirect vaccination requirement. You can only go to concerts if you have a vaccination certificate. (…) That sounds to me like the Jewish star, clearly, if you don’t have a Jewish star, you can go there and there, and if you have one, you have to stay at home. So that’s already, well, I’m comparing the time. Of course, what happened under National Socialism was much, much worse, that‘s for sure. And the atrocities are much worse there, but the beginnings are exactly the same.” (R1).

In her statement she is aware of the possibility that this HA might backfire for certain people, and qualifies it by saying “the atrocities are much worse there, but the beginnings are exactly the same”. In this we see that even those that are deeply committed to a particular HA can reflect on and nuance it for more strategic rhetorical deployment.

Her general concern is, like the above, how vaccines fit into a government program of surveillance and exclusion. She even entertains the possibility of vaccine microchips but does not fully accept the premise. Part of her argument against vaccines draws on a HA to the Swine Flu vaccine scandal, and according to her, people are already realising COVID-19 vaccinations do not work and themselves cause medical problems. The distrust in medical research also expresses itself in her belief that the coronavirus probably comes from a lab and is a biological weapon:

There are several rumours that it probably comes from the laboratory. And I could also imagine, because it is a security level 4 laboratory… and there is always research being done on biological warfare agents, I would say. It’s not for nothing that the French president said: “We are (…) at war”. (…) Unfortunately, I think humanity is capable of everything. (…) You wouldn’t have thought that Hitler would do all that or that people would go along with it. (…) I really think humanity is capable of great atrocities.

This statement was made when the lab leak theory of the coronavirus’s origins was considered a fringe conspiracy theory. Different intelligence agencies have since released reports that see it as one possible explanation while recognizing we will probably never know the truth—fertile ground for future conspiracy theories! While other participants also entertained the possibility of the coronavirus being leaked from a lab leak, what is notable in the present account of it is that the claim is partly justified through a HA to Hitler’s atrocities which was unbelievable at the time. Moreover, she misuses Macron’s metaphorical statement about being at war with COVID-19 to suggest it implied a literal act of war.

## Conclusion

This study has gone beyond the current state of the art by systematically exploring how HAs were employed in everyday thinking through the COVID-19 pandemic. Using a three-step analytic framework, we identified which analogies were used, tracked their evolution over time, characterized their frequent users, and closely examined their personal psychological functions through a detailed case study. Our findings reveal that the overall use of HAs decreased as the pandemic progressed, with medical analogies initially dominant but later overtaken by political analogies. This shift reflects the continued relevance of political analogies, particularly for individuals resistant to government-imposed measures.

We further demonstrated a clear division in HA use between societal majorities (who largely accepted official narratives and complied with pandemic measures) and active minorities (who challenged, criticized, or even protested these measures). Contrary to portrayals of dissenters as passive recipients of misinformation, participants in our study often demonstrated higher informational engagement than their counterparts in the majority. For these individuals, HAs served as crucial rhetorical tools for articulating resistance in the face of normative societal pressures (Moscovici, 1985). This group saw their own efforts as critically engaging with different sources and drawing connections between events, although from a standpoint of extreme distrust in government and mainstream institutions, including mainstream media. While it is accurate to characterize these frequent HA users as exhibiting high psychological reactance (Brehm & Brehm, 1981)—resistance to perceived threats to their autonomy—our findings highlight the equally important role played by long-standing issues of institutional distrust and identifications with minority positions.

The study uncovered a nuanced and notable relationship between frequent HA usage and conspiracy thinking. Participants who extensively employed HA displayed significantly higher levels of conspiracy mentality (Bruder et al., 2013). Importantly, this relationship is not simplistic or unidirectional: while benign historical references, such as the 1918 pandemic, do not inherently foster this kind of suspicion, HAs involving sinister governmental actions or medical scandals clearly promote and reinforce a conspiratorial worldview. These types of analogies recall past breaches of public trust and legitimize contemporary skepticism toward authorities. For instance, historical medical abuses—such as the Tuskegee syphilis experiment, which continues to fuel skepticism about public health interventions among African Americans (Zekeri, 2024)—highlight how singular events can have lasting impacts across generations. In our own sample, references to medical scandals like the Swine Flu vaccine were strategically deployed to cast doubt on COVID-19 vaccination efforts and accuse the government of withholding critical information. Political HAs, particularly those involving Germany’s experiences with National Socialism and the GDR, were used more widely and in relation to a broader range of issues. They are a deep-rooted presence in German collective memory, and continue to influence contemporary attitudes toward issues such as digital surveillance and personal liberties.

Our findings align with Moscovici’s (2020) conception of conspiracy theories as anchored in an *Urphänomen*—a generative cause that recurs through history. For example, Jews were once accused of poisoning wells and now blamed for ‘space lasers’. There were 3G, then 4G and now 5G conspiracies (though 5G conspiracies were rare in our Germany sample, probably due to the poor 5G coverage in the country!). HAs explicitly connect contemporary suspicions to historical distrust, thus making previously implicit conspiracy narratives explicit: “They did it before; they are doing it again now”—the “they” here being politicians, medical institutions or other powerful actors. Conspiracy theories typically proliferate during periods of crisis and societal upheaval, serving to create coherent counter-narratives (van Prooijen & Douglas, 2017). However, existing psychological literature frequently overlooks the deeply personal aspects of this phenomenon, beyond it being motivated by anxiety. The case study analyzed in this paper explicitly addressed this gap by illustrating how HAs fulfill deeply personal psychological needs. Specifically, we propose extending Ghilani et al.’s (2017) framework to include a “personal psychological function,” highlighting how HA can speak to deep and affective life tensions—existential and relational—and provide a symbol for their resolution.

In our detailed case study, the participant’s consistent invocation of Nazi-era HAs was not merely an interpretative frame but a profound expression of family memory, a tradition of resistance, and affective bonds—particularly exemplified by the symbolic role of her grandfather. Despite previous research suggesting a rewriting of her grandfather’s historical role (Welzer, 2005), framing him as a resistance hero reinforced her own identity as a critical thinker and justified her actions against perceived contemporary oppression. With this move she brings together a *macro* level (historical representations of the Nazi past), a *meso* level (family memory of resistance) and *micro* level (personal struggles during the pandemic) (Cordonnier et al., 2022). The Nazi HA served as a protective lens through which all pandemic-related events were filtered. This aligns closely with Adorno and Brunswik’s (1950) concept of ‘intolerance to ambiguity,’ wherein rigid frames provide psychological stability amid uncertainty, which has also been used as an explanation of conspiracy thinking. Her ability to participate alongside neo-Nazi protesters while simultaneously believing herself to be combating fascism further underscores the psychological depth and complexity of such HAs. Rather than experiencing cognitive dissonance, the overarching analogy of fighting oppression allowed her to resolve and rationalize contradictions, demonstrating the profound power of HAs as psychological tools in navigating personal, social, and political turmoil.

## Data Availability

The complete dataset, including interview transcripts (in German), SPSS demographic files, and the full interview guides in German and English, is openly accessible via Zenodo: [10.5281/zenodo.5556052] (10.5281/zenodo.5556052)

## Appendix

**Table 4:** Keyword List applied to search for historical analogies in the text

German search term	English tranlsation/Explanation
Pest	Plague
Spanische Grippe	Spanish Flu
1918	Year marking the end of WWI and start of the Weimar Republic
1919	Treaty of Versailles and post-WWI conditions
1933	Hitler’s rise to power/start of Nazi dictatorship
Weltkrieg	World War
Krieg	War
Nationalsozialismus – National Socialism	
Nazi	Nazi
Hitler	Hitler
DDR	German Democratic Republic (East Germany)
Flüchtling	Refugee (e.g., in reference to the 2015 refugee crisis)
SARS	Severe Acute Respiratory Syndrome (2002–2003)
Schweinegrippe	Swine Flu (2009)
Vogelgrippe	Avian Flu/Bird Flu
Ebola	Ebola outbreaks
Contergan	Thalidomide scandal (late 1950s–early 1960 s)
Skandal	Scandal
Geschichte	History
Damals	Back then/In the past
Gelernt	Learned (as in “we have learned from history”)
